# Gender-based violence among healthcare students: Prevalence, description and associated factors

**DOI:** 10.1371/journal.pone.0288855

**Published:** 2023-11-30

**Authors:** Marie-Pierre Tavolacci, Alice Karmaly, Najla El Gharbi-Hamza, Benoit Veber, Joel Ladner

**Affiliations:** 1 Univ Rouen Normandie, UMR1073 ADEN, CHU Rouen, CIC-CRB 1404, Rouen, France; 2 Department of Physiotherapy, ERFPS-CHU Rouen, Rouen, France; 3 Department of Pharmacy, Univ Rouen Normandie, Rouen, France; 4 Surgical Intensive Care Unit, Univ Rouen Normandie Health Campus, CHU Rouen, Rouen, France; 5 Department of Epidemiology and Health Promotion, Univ Rouen Normandie, UMR1073, CHU Rouen, Rouen, France; Nigerian Institute of Medical Research, NIGERIA

## Abstract

**Objectives:**

The aims of the current study were 1) to provide the prevalence of five types of gender-based violence (GBV) among male and female healthcare students; 2) to describe perpetrators’ status, where the GBV occurred, and psychological and behavioural impacts of the GBV; and 3) to identify factors associated with GBV.

**Design:**

A cross-sectional study was conducted among voluntary healthcare students in France.

**Setting:**

Health Campus at Rouen and nursing schools in Normandy, France.

**Participants:**

Volunteer healthcare students of 18 years and over.

**Data collected:**

Five types of GBV were recorded: GBV1: damage to a person’s image due to a sexual connotation on social networks, GBV2: sexist remarks and behaviour, GBV3: comments with sexual connotations, GBV4: sexual assaults and GBV5: rape or attempted rape. Perpetrators’ status, where the GBV occurred, psychological and behavioural impacts of GBV were also recorded.

**Results:**

One thousand one hundred and fifty-two students were included. The mean age was 20.8 years (SD = 2.26), 82.6% of students were women (0.4% non-binary). Since the beginning of their healthcare study, 41.2% of students CI 95% [39.7–42.6] were victim of at least one type of GBV: 15.8% among men CI 95% [13.2–18.4] and 46.0% CI 95% [44.4–47.6] among women (p < 0.001). The perpetrators were student peers, regardless of the type of GBV, healthcare workers for the GB2 and GBV3, and patients except for GBV5. The main consequences of GBV on health was psychological and eating related. After multivariate analysis, being a woman, LGBTQ+ (Lesbian, Gay, Bi, Trans, Queer and other), a nurse, a physiotherapist student, having a job, living with roommates and year of study were risk factors for GBV.

**Conclusion:**

GBV is so common at healthcare universities and could have such severe consequences for students that more work is needed to generate a culture change and ensure safe learning environments.

## Introduction

The United Nations’ definition of gender-based violence (GBV) is “any act of gender-based violence that results in, or is likely to result in, physical, sexual or psychological harm or suffering to women whether occurring in public or private life. GBV is a somewhat more inclusive term than violence against women. GBV can include violence against men provided the violence stems from a man’s gender identity or presentation [1]. Sexual and gender-based violence is any act committed against a person’s will and is based on the different roles that society assigns to men and women, and on unequal power relations. It can be physical, emotional, psychosocial and sexual in nature.

GBV clearly exists on college/university campuses as documented by both social phenomena and research. Prevalence rates have varied greatly, mainly due to differences in definitions, the reporting period measured, sampling strategies, and the measures used [2]. Studies measured different forms of sexual victimisation separately (e.g. forcible rape, incapacitated rape, unwanted sexual contact, etc.); however, some studies measured multiple forms of victimisation in one item. The most common forms of lifetime (ever having experienced) sexual harassment were”sexual expressions, suggestions or comments about your body” and”unwanted touching, hugging or kissing” (both 15.4%), while rape and rape attempt were reported by 3.4% and 2.1%, respectively [3]. Among college students, regarding unwanted sexual contact (excluding rape or attempted rape), the majority of studies reported estimates of over 20% among college women, although the rates ranged widely from 1.8% to 34% [2]. The particularity of health students is that the strong hierarchical relationships and patriarchal history, the long hours of service, the high levels of tension and the many opportunities for intimacy promote sexual violence. The first major study among medical students was conducted in the early 1990s [4]. Female medical students were twice more likely than non-science, technology, engineering and math majors to experience sexual harassment by faculty or staff [5]. A meta-analysis reported a prevalence for harassment and discrimination during undergraduate medical training and clerkship of 59.6% [6]. The majority of studies focused predominantly on white, female undergraduate students [7, 8]. Studies in France are sparse; one study was conducted among all the curricula in 2020: 5% of female students had been a victim of rape, and 10% of female students had been a victim of sexual violence [9]. Few studies were conducted among healthcare students and mainly medical students [10, 11]. It is not known whether health study fields are particularly at risk of GBV. The French minister of higher education and research promotes GBV research among students in 2021 [12]. Sexual harassment is considered offensive by the recipient and as exceeding one’s coping mechanisms, it can also be felt tothreat one’s well-being in that the victim finds it difficult to cope with or to handle. Sexually harassed students have been shown to be more likely to engage in risky behaviours, such as increased drug use, problematic drinking behaviours, sexual risk-taking and sexual dysfunction [13–15]. Students who experience GBV are more likely to skip class, get lower grades, switch programs or drop out altogether [16]. Additionally, students’ social circumstances and trauma exposures, such as witnessing or experiencing interpersonal violence or violent crime or rape, all potentially leading to grievous bodily harm, increase the risk for depressive symptoms, PTSD and suicidality. The potential future risk is that when these students become health professionals, they will have lower mental health, job satisfaction and sense of safety at work [17].

The aims of the study were as follows: 1) to provide the prevalence of five types of GBV among male and female healthcare students; 2) to describe perpetrators’ status, where the GBV occurred, and the psychological and behavioural impacts of the GBV; and 3) to identify factors associated with GBV.

## Methods

### Study design

Healthcare students participated voluntarily in an online cross-sectional study at the Health Campus at Rouen and at nursing schools in Normandy, France. The questionnaire was pretested by five health students and residents and improved according to feed-back. All students (n = 4540) were invited to complete an anonymous self-administered electronic questionnaire sent via the students’ university e-mail list with two e-mail reminders. Others invitation were also used: social networks of university’ students associations (e.g. on Facebook), and posters placed at strategic sites at the university. The recruitment period lasted from 1st October, 2022 to 30th November, 2022.

Students were eligible for inclusion if they were currently enrolled in a higher education health curriculum and were aged 18 years or older. Students over 30 were secondarily excluded.

### Ethics approval and consent to participate

The Rouen University Hospital’s Institutional Review Board (IRB) approved the observational study design and waived the need for informed consent (E-2022-56). All methods were performed in accordance with the Helsinki declaration.

## Data collection

### Socio-economic characteristics

Data on age, gender, grant holder status, student job status, accommodation status (domiciled at parents, with a roommate, in couple or alone) and sexual orientation (LGBTQ+(Lesbian, Gay, Bi, Trans, Queer and other) or not)) were collected. Data on the health program (nursing, medical, pharmacy, midwife, physiotherapy, speech therapy and others) and the academic year of study were also collected. Depression was assessed using the eight items of the CESD-8 (Center for Epidemiologic Studies-Depression) scale. The response values were scored on a 4-point Likert scale (range 1 to 4) and CESD-8 on a scale from 8 to 32, with higher scores indicating a higher frequency of depressive complaints [18].

### Gender-based violence

Five types of GBV were collected: GBV1: damage to a person’s image due to a sexual connotation on social networks (sextape, sexto, sexfie), GBV2: sexist comments s and behaviour based on a person’s gender, such as remarks and comments about a person’s physical appearance, about skills related to the person’s gender that are intended to undermine their dignity or create an intimidating, hostile, degrading or humiliating environment; GBV3: comments with sexual connotations: dirty jokes, lewd remarks relating to sex, breasts, buttocks, and, sexuality, GBV4: sexual assaults such as hand on buttocks, thighs, sex, breasts, kiss by surprise, coercion or threat and GBV5: rape or attempted rape such as coerced sex, penetration without consent, forced oral sex [19, 20]. If the student reported having been a victim of one of these GBVs, then more information was collected about the GBV such as how many timed it happened, the gender and status of the perpetrators (healthcare workers, teacher, peer students, patients, the location of the GBV (at the hospital, university, during a party or at a private location).

### Impact of gender-based violence

Student victims of GBV declared the perceived impact on their mental health (psychological, sleep-related, academic, friendship and love life) and health behaviours (eating-related, alcohol, tobacco, anxiolytic/antidepressant, cannabis/drugs consumption) from none (1) to major impact (5). Post-traumatic stress disorder (PTSD) was assessed using the twenty items of the PCL-5 (post-traumatic stress disorder checklist for DSM-5) scale [21]. The response values were scored on a 5-point Likert scale (range 0 to 5) and PCL-5 range from 0 to 80: score 32 to 36: probable PTSD; 37 and more: certain PTSD.

### Statistical analysis

A sampling weighting was performed to ensure a representative sample (annex). Answers were mandatory, thus there are no missing data. Qualitative variables were summarized by percentage and compared using the Chi2. test and continuous variables by mean with standard deviation (SD) and compared using the Student Test. C Multivariable logistic regression model was performed to identify factors associated with GBV. Variables with a p < 0.20 in univariate analysis were entered in the model. For each health behavior, adjusted odds ratios (aOR) are provided with their 95% confidence interval (95%CI).

### Public involvement statement

Three healthcare students participated in the creation of the questionnaire, its dissemination and the results via social networks.

## Results

**Fig 1:**
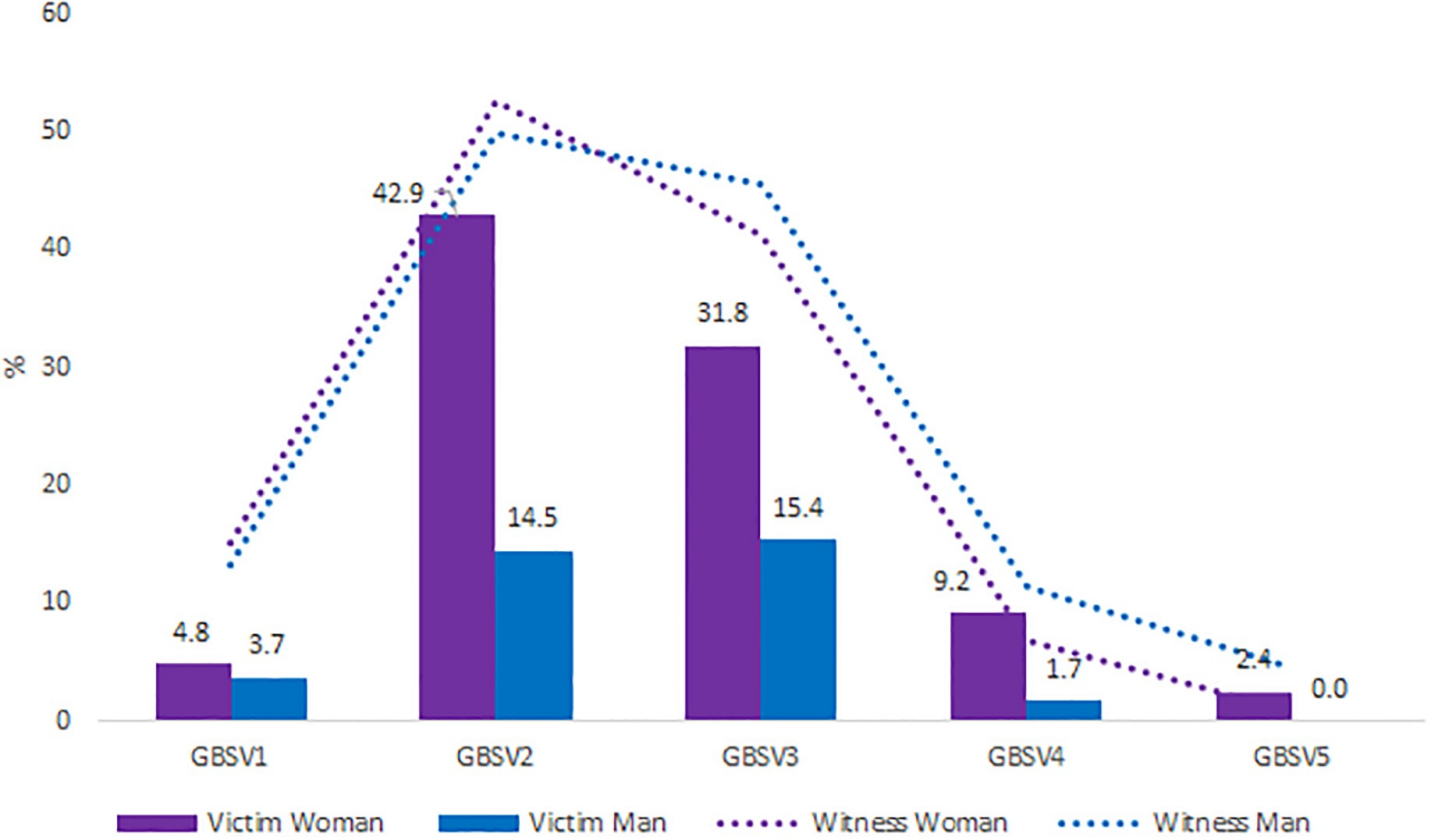
Prevalence of health student’s victim or witness of gender based violence according to the gender.

**Fig 2:**
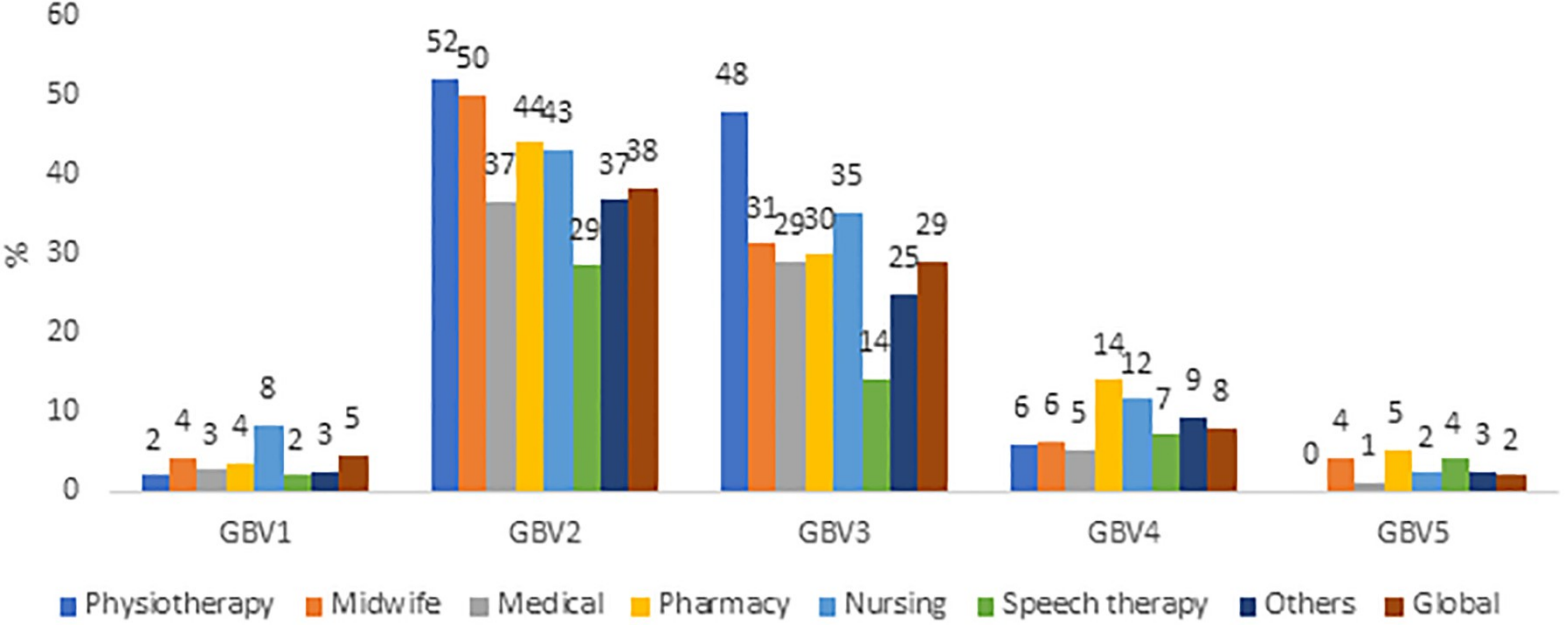
Prevalence of health student’s victim of gender based violence according to the curriculum.

**Fig 3:**
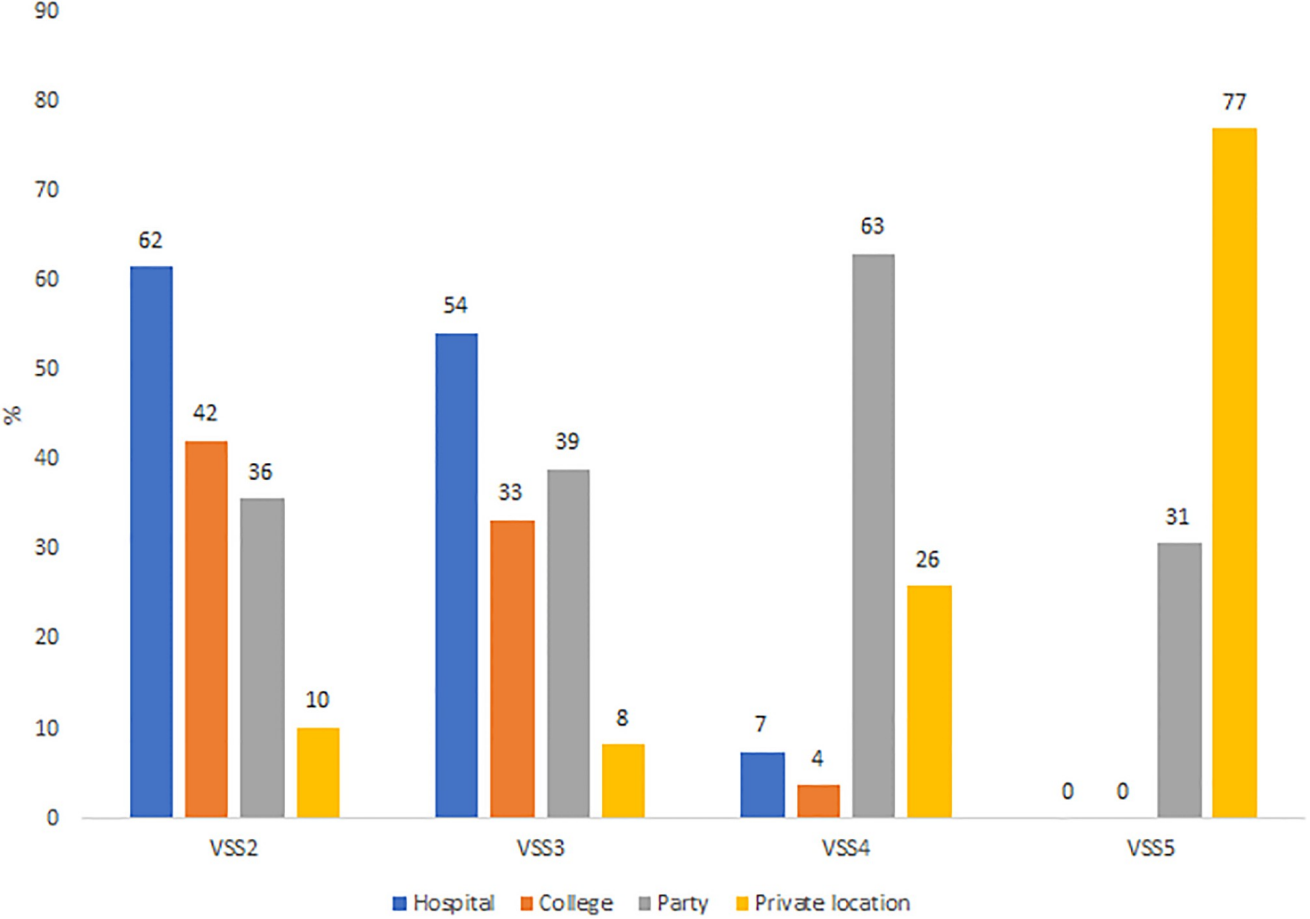
Gender based violence location. *total by GBV could be over 100% because students could have been victim of more than one GBV.

**Fig 4:**
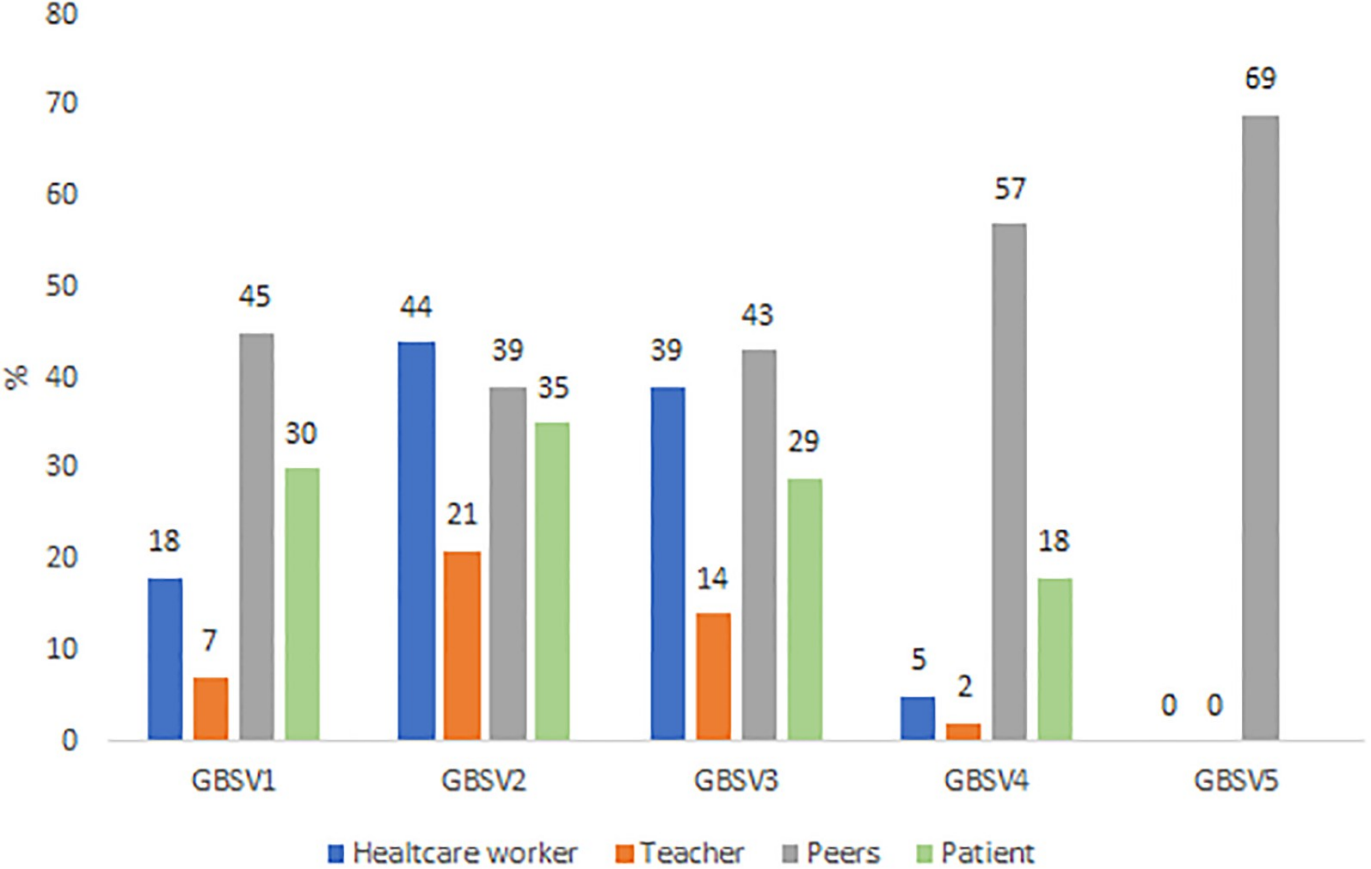
Perpetrators status of gender based violence. *total by GBV could be over 100% because students could have been victim of more than one GBV.

**Fig 5:**
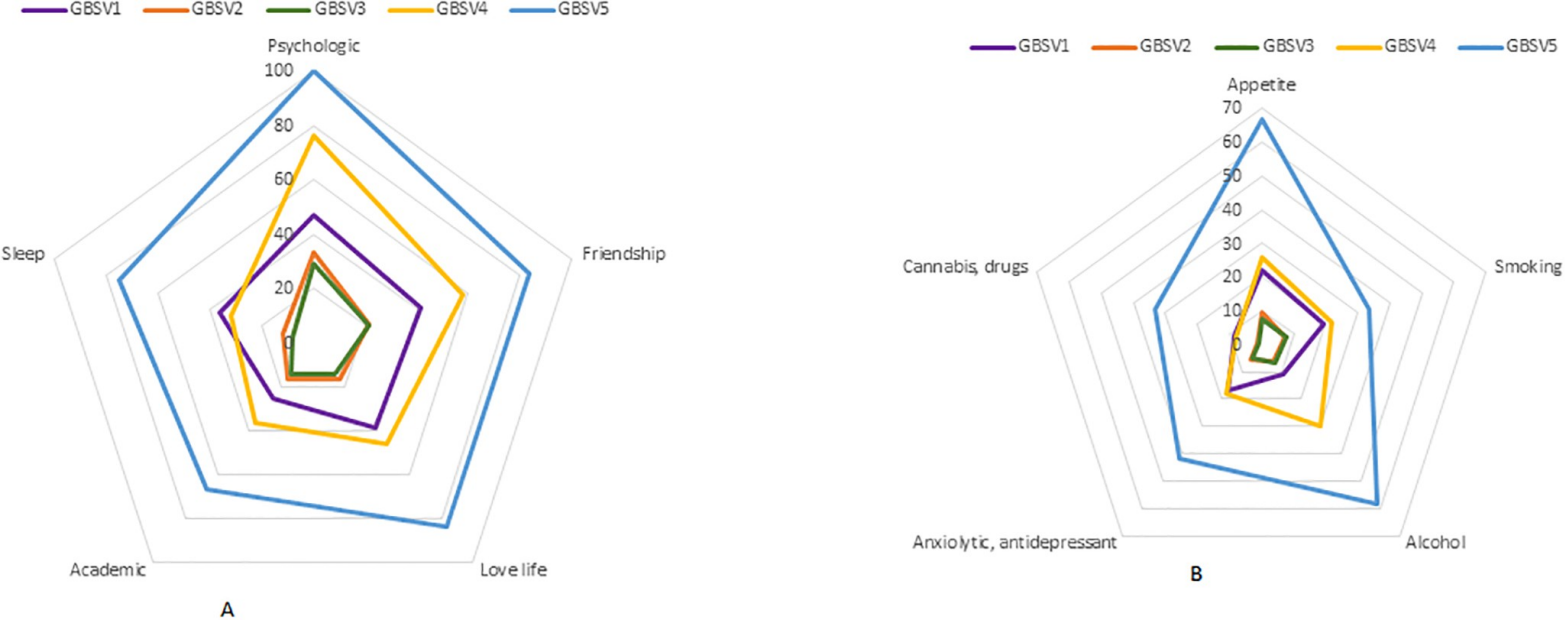
Gender based violence impact (3 medium to 5 major): Mental health/wellness (A) and behaviors (B).

Overall, 1152 students were included with a participation rate of 25.3%. The mean age was 20.8 years (SD = 2.26), 82.6% of students were women (0.4% non-binary), and 74.6% were in the first three years of study. Socio-demographics characteristics are described in Table 1. Since the beginning of their chosen health curriculum, 41.2% of students CI 95% [39.7–42.6] were victim of at least one type of GBV: 15.8% among men CI 95% [13.2–18.4] and 46.0% CI 95% [44.4–47.6] among women (p < 0.001). Of these students, half have been victim to multiple GBVs. The most frequent type of GBV was GBV2 (sexist comments): 14.5% among male students and 42.0% among female students. The prevalence by gender of victims are displayed in Fig 1. Prevalence by curriculum were: 56.0% in the physiotherapist,50.0% in midwife, 39.0% in medicine, 47.4% in pharmacy, 46.3% in nursing and 32.3% in other curricula. The prevalence of each GBV according to the curriculum were displayed in Fig 2. All types of GBV occurred during parties (mainly GBV4: 63%), hospitals and colleges were the places where GBV2 (62% and 42%) and GBV3 occurred (54% and 33%), aand GBV5 occurred in private places (77%) (Fig 3). Fig 4 displays the status of the perpetrators of GBV. Peer students were the main perpetrators of GBV mostly for GBV1 (45%),4 (57%) and 5 (69%), healthcare workers for GBV2 (44%) and 3 (39%), teachers for GBV2 (21%) and patients for GBV1 (30%), 2 (35%) and 3 (29%). Certain and probable PTSD occurred respectively for 10.5% and 4.9% of the victims of GBV. The impact of GBV on mental health reported by students are described in Fig 5A: Sexual violence (GBV4 and GBV5) were the GBVs with the greatest mental health impact. Whatever the GBV, psychological impact was the main mental health impact (29% for GBV3 to 100% for GBV5). Fig 5B shows the perceived impact on health behaviour: impact on eating related was the main impact on health behaviour (7.7% for GBV3 to 67% for GBV5). Major consequences on health behaviour occured forGBV5. After multivariate analysis, being a woman, LGBTQ+, a nurse, a physiotherapist students, having a job, living with roommates and year of study, were significant risk factors of GBV (Table 1).

**Table 1:** Factors associated to be victim of gender based violence among health students (univariate and multivariate analysis) N = 1152.

	GBV (n = 461)	No GBV (n = 691)	Total (N = 1152)	p	AOR CI95%
Age (SD°	21.0 (2.08)	20.6 (2.36)	20.8 (2.26)	0.006	1.11 (1-04-1.18)
Gender % (n)				<0.0001	
Male	5.4 (25)	24.6 (170)	16.9 (195)		Ref
Female	93.7 (432)	75.2 (520)	82.6 (952)		6.72 (4.21–10.72)
Academic Year of study % (n)				<0.0001	
1	18.7 (86)	30.1 (208)	25.5 (294)		Ref
2	24.1 (111)	24 (166)	24.1 (277)		1.76 (1.14–2.72)
3	24.7 (114)	25.2 (174)	25 (288)		1.58 (1.01–2.44)
4	13 (60)	9.5 (66)	10.9 (126)		2.87 (1.57–5.24)
5	10.8 (50)	6.1 (42)	8 (92)		4.24 (2.18–8.24)
6	8.7 (40)	5.1 (35)	6.5 (75)		5.25 (2.40–11.49)
Health Curriculum % (n)				<0.0001	
Physiotherapist	6.1 (28)	3.2 (22)	4.3 (50)		2.17 (1.08–4.35)
Midfwife	5.2 (24)	3.5 (24)	4.2 (48)		1.08 (0.55–2.13)
Medecine	37.5 (173)	39.2 (271)	38.5 (444)		0.98 (0.65–1.17)
Pharmacy	5.9 (27)	4.3 (30)	4.9 (57)		1.13 (0.57–2.24)
Nursing	21.7 (100)	16.8 (116)	18.7 (216)		2.05 (1.39–3.03)
Other	24.6	33.0	29.4		Ref
Accommodation status % (n)				0.007	
Domiciled at parents	26.5 (122)	34.3 (237)	31.2 (359)		Ref
With roommates	13.7 (63)	8.5 (59)	10.6 (122)		1.93 (1.22–3.07)
In couple	14.7 (68)	14.5 (100)	14.5 (168)		0.95 (0.61–1.50)
Alone	45.1(208)	42.7 (295)	43.7 (503)		1.21 (0.88–1.67)
LGBTQ+ % (n)	19.3 (89)	11.7 (81)	14.8 (170)	<0.0004	2.13 (1.48–3.06)
Job % (n)	33 (152)	25.2 (174)	28.3 (326)	0.004	1.38 (1.2–1.87)
Grant % (n)	31.5 (145)	28.4 (196)	29.6 (341)	0.26	
CESD-8 mean (SD)	15.7 (4.7)	15.4 (4.9)	15.5 (1.8)	0.25	

SD: Standard Deviation

GBV1: damage to a person’s image due to a sexual connotation on social networks

GBV2: sexist remarks and behaviour, comments or behavior based on a person’s gender, GBV3: comments with sexual connotations

GBV4: sexual assault

GBV5: rape or attempted rape

## Discussion

Our sampling weighting brought to light that 41.2% of healthcare students have been exposed to at least one GBV since the beginning of their health studies. Although comparisons between studies are difficult due to methodological differences (definition, prevalence period, curricula). Our study shows that healthcare students do not appear to be more victimised than other students. Indeed in a large recent study in France, 10% of students had been sexually assaulted, and 5% had been raped, 60% had been victims of whatever GBV [22]. As can be seeen in the meta-analysis [6], we also reported sexist language or sexual comments were the most commonly cited forms of GBV., Among French pharmacy, dental and medical students, sexual aggression occurred in 5.5%,n, which is consistent with our study [11]. Moreover, appropriate identification of a violent situation (sexual or psychological) is highly variable across students and cultures, with under-recognition of inappropriate situations ranging from 15% to 50% of cases [23]. Our study provides details about the authors of GBV, with specific reference to the healthcare environment. The perpetrators were student peers, regardless of the type of GBV, healthcare workers were responsible for the sexist and sexual verbal comments as reported in Canada [24], and were patients except for rape, (this also reported by Mahurin et al. [25]). We also highlighted that GBV related to sexist and sexual verbal comments occurred mainly at the hospital which is a particularity of health students compared to other students. Sexual assault and rape occurred in private location which can be explained by factors related to a campus party culture (e.g. binge drinking, alcohol use, drug use [26]).

The impacts of GBV on university students must be considered. Indeed, in our study 15.4% of students have PTSD related to GBV, as reported in another study among medical students [27]. We did not find a difference in the depression score between students who had been exposed students who had not been non-exposed to a GBV while Rolland had shown a higher risk of major depressive episode associated with GBV [24]. Psychological and relationship impacts were consequences particularly for online sexual aggression and rape, which should not be overlooked because it could lead to suicide [28]. Impacts were not only psychological but also branched out into health behaviours (mainly alcohol and drugs consumption) as reported among women students [29]. Importantly, the use of alcohol as a potential coping mechanism following a sexual assault, consistent with the self-medication hypothesis, could create a cyclical experience for women that increases the risk of future assaults [30]. As reported by a meta-analysis, women are more likely to experience GBV than men [26]. Most studies focus on medical students and not on all health students; therefore, our study provides new knowledge that nursing and physiotherapy students are the most at risk for GBV. As expected and relevant for the literature, the risk of GBV increases with the number of years of study [28]. Being LGBTQ+ increases the risk of experiencing GBV among healthcare students as also reported among students in all areas of study [31, 32]. It could be important to raise awareness among healthcare workers and universities regarding sexism and homophobia and to be more inclusive LGBTQ+ people.

### Limitations

Caution is advised when generalizing these findings, for the following reasons. First, it was a convenience sample, voluntary participation could have led to representativeness and self-selection. We limited this bias with a weighting sample however extrapolation to all health students in France must be done with precaution. Second although the definition of each GBV was specified in the questionnaire it is possible that students perception of what constitutes GBV is different which could lead to an information bias.

### Future directions

GBV is so common among healthcare students and can have such severe consequences for students that more work is needed to generate a culture change and ensure safe learning in university and hospital. In a French school of medicine, Lisan et al. showed that the implementation of such an ‘observatory of mistreatment’ may in itself contribute to reducing both sexual harassment and psychological abuse [33]. Supportive services and efforts to address peer cultures that promote harassment are needed: integrate student associations and representatives fully in the design, implementation and monitoring of policies to combat GBV should be considered. Given the potential consequences suffered by those exposed to GBV, both the institutions and student welfare organisations should intensify practices and policies to prevent and respond to GBV among university students. To help students recognise GBV and learn how to intervene in these situations, more GBV information is needed in prevention education programs that integrates content pertinent to victimisation risk, perpetration risk and bystander intervention skills [34]. Johnson et al. suggested that a semester-long course targeting first-year students could potentially influence knowledge, attitudes and behavioural intentions regarding sexual violence and create a more positive campus climate [35, 36]. Additonal programs that depict victims and perpetrators in a gender-neutral manner had more favorable effects on bystander efficacy than programs that depicted victims as all or mostly women or perpetrators as all or mostly men, respectively [37].

## Supporting information

S1 Checklist STROBE statement—Checklist of items that should be included in reports of *cross-sectional studies*. (DOCX)
